# cGMP-mediated antioxidant signaling: a role for the c-Abl tyrosine kinase

**DOI:** 10.1186/1471-2210-11-S1-P70

**Published:** 2011-08-01

**Authors:** Robert S Stephens, Laura E Servinsky, David B Pearse

**Affiliations:** 1 Division of Pulmonary and Critical Care Medicine, Johns Hopkins University, Baltimore, USA

## Background

Oxidant injury to the pulmonary endothelium contributes to acute lung injury. We have shown that activation of PKG_I_ by cGMP increases protein levels of the antioxidant enzymes catalase and glutathione peroxidase-1 (GPx-1) and ameliorates oxidant injury in mouse lung endothelium [1]. Catalase and GPx-1 mRNA was not increased. The pathway downstream of PKG_I_ that leads to increases in catalase and GPx-1 is unknown. The c-Abl tyrosine kinase has been reported to regulate catalase and GPx-1; fibroblasts deficient in c-Abl have increased levels of catalase and resist oxidant injury [2-4]. We hypothesized that 1) activation of PKG_I_ would decrease c-Abl protein levels; and 2) inhibition of c-Abl with imatinib would increase antioxidant proteins and hydrogen peroxide (H_2_O_2_) degradation, attenuate H_2_O_2_-induced endothelial permeability, and decrease H_2_O_2_-induced cell death in mouse lung microvascular endothelial cells (MLVMEC).

## Methods

MLMVEC were isolated from wild-type (wt) and PKG_I_ knock-out (PKG_I_ -/-) C57Bl/6 mice. MLMVEC were treated for 4 hours with 8pCPT-cGMP (50 μM) or imatinib. H_2_O_2_ scavenging was measured with a H_2_O_2_ electrode after addition of known concentrations of H_2_O_2_ to cells in suspension. Nuclear condensation was assessed using fluorescence microscopy. Transendothelial resistance (TER) was measured using electric cell-substrate impedance sensing (ECIS). Proteins were quantified by Western blotting.

## Results

Treatment of wt MLMVEC with 8pCPT-cGMP significantly decreased protein levels of c-Abl by 29% (p=0.02). 8pCPT-cGMP had no effect on cAbl in PKG_I_ -/- MLMVEC. 7 days of sildenafil treatment (100mg/kg/day) [5] significantly decreased whole lung c-Abl protein by 37 % and increased whole lung catalase protein by 40% (p<0.05 for both). Treatment of wt MLMVEC with 10 and 20 µM imatinib increased catalase protein by 16% and 37% respectively, and GPx-1 protein levels by 24% and 36%, respectively (p<0.05 for all values). Imatinib (10 µM) decreased the measured peak H_2_O_2_ after addition of extracellular H_2_O_2_ (20, 50, and 100 µM) by 18%, 23%, and 9%, respectively (p ≤0.05 at all concentrations). Imatinib (10 µM) significantly attenuated 100 and 250 µM H_2_O_2_-induced nuclear condensation by 15% (p<0.05). Finally, imatinib (20 µM) attenuated the H_2_O_2_-induced TER decrease in monolayers of MLMVEC (p<0.05 by ANOVA).

## Conclusion

These data suggest that cGMP, through PKG_I_, increases MLMVEC antioxidant activity by down regulating the cAbl tyrosine kinase. Inhibition of c-Abl with imatinib attenuates oxidant-induced MLMVEC death and dysfunction, mimicking the antioxidant effects of cGMP-PKG_I_ signalling. The mechanism linking activated PKG_I_ and cAbl expression is unknown.
